# Heuristic Method for Minimizing Model Size of CNN by Combining Multiple Pruning Techniques

**DOI:** 10.3390/s22155874

**Published:** 2022-08-05

**Authors:** Danhe Tian, Shinichi Yamagiwa, Koichi Wada

**Affiliations:** 1 Doctoral Program in Computer Science, University of Tsukuba, 1-1-1 Tennodai, Tsukuba 305-8573, Ibaraki, Japan; 2 JST, SPRING, 4-1-8 Honcho, Kawaguchi 332-0012, Saitama, Japan; 3 Faculty of Engineering, Information and Systems, University of Tsukuba, 1-1-1 Tennodai, Tsukuba 305-8573, Ibaraki, Japan; 4 JST, PRESTO, 4-1-8 Honcho, Kawaguchi 332-0012, Saitama, Japan

**Keywords:** deep learning, convolutional neural network, network pruning, object detection

## Abstract

Network pruning techniques have been widely used for compressing computational and memory intensive deep learning models through removing redundant components of the model. According to the pruning granularity, network pruning can be categorized into structured and unstructured methods. The structured pruning removes the large components in a model such as channels or layers, which might reduce the accuracy. The unstructured pruning directly removes mainly the parameters in a model as well as the redundant channels or layers, which might result in an inadequate pruning. To address the limitations of the pruning methods, this paper proposes a heuristic method for minimizing model size. This paper implements an algorithm to combine both the structured and the unstructured pruning methods while maintaining the target accuracy that is configured by its application. We use network slimming for the structured pruning method and deep compression for the unstructured one. Our method achieves a higher compression ratio than the case when the individual pruning method is applied. To show the effectiveness of our proposed method, this paper evaluates our proposed method with actual state-of-the-art CNN models of VGGNet, ResNet and DenseNet under the CIFAR-10 dataset. This paper discusses the performance of the proposed method with the cases of individual usage of the structured and unstructured pruning methods and then proves that our method achieves better performance with higher compression ratio. In the best case of the VGGNet, our method results in a 13× reduction ratio in the model size, and also gives a 15× reduction ratio regarding the pruning time compared with the brute-force search method.

## 1. Introduction

Convolutional neural network (CNN) models have been rapidly growing since their appearance in 1990 [1]. Due to their good performances of feature detection availability, during the past decades, CNN models have been widely applied in a range of various fields such as natural language processing [2], autonomous driving systems [3] and computer vision. Especially in computer vision applications with examples including object detection [4,5,6], image classification and recognition [7,8,9,10], image segmentation [11,12], and human pose estimation [13,14], the CNN models show enormous influence on computer vision fields such as industrial, society and academic applications.

To achieve higher precision, the CNN models always have a wider and deeper architecture. Some of the new state-of-the-art models have over millions or even trillions of parameters. This inevitably raises the enormous needs of massive computations, large energy consumption and huge memory/computational resources [15,16]. According to these requirements, CNN-based applications need to challenge availability for their implementation on edge devices where the amount of resources are constrained [17,18] such as smartphones or vehicles. Therefore, it is essential not only to shrink and lighten the CNN models, but also to maintain their high accuracy to meet the growing demands in real-world applications.

Recently, extensive works have been proposed to compress these large-scale models. Network pruning is one of the most well-known techniques and attracts enormous attention. It enables huge CNN models to run efficiently on edge devices by removing superfluous parts of the models. According to the pruning granularity, network pruning can be roughly categorized as structured pruning [19,20] and unstructured pruning [21,22]. The structured pruning removes the larger structures of the CNN models such as channels or layers. This results in a direct reduction of the computational load, especially for those computation-intensive convolutional layers. However, directly removing channels or layers entirely may also result in lower accuracy for the inference. On the other hand, the unstructured pruning compresses the CNN models by removing the individual unimportant weights.

In this paper, we propose a heuristic method that combines the structured and unstructured pruning methods. In our method, we control the compression ratios of the original structured and unstructured pruning methods by repeating these methods under a target accuracy. Through combining these two pruning methods, we will explore the effective pruned model with a higher compression ratio than the single usage of them, respectively, and simultaneously achieve higher accuracy for the inference.

The main contributions of this paper are summarized as follows:

1. We have found a method for model compression that uses the structured pruning and unstructured pruning jointly. We also allow the CNN models to meet and maintain the target accuracy given by their applications. This will achieve the best reduction ratio regarding the parameters of the CNN models.
2. We have developed an algorithm that achieves a better compression ratio than the individual usage of the structured and unstructured pruning methods under a target accuracy.
3. We have shown the efficiency of our proposed method according to evaluations with five actual CNN models and validated the correctness of the algorithm.
4. We also optimized our proposed algorithm to require significantly less computational time to find the best compression ratio compared to the case when we apply brute-force search algorithm.

The rest of this paper is organized as follows. The next section introduces the background and the definitions of this research focusing on the object detection techniques with CNN and the pruning techniques to compress the network model. Section 3 explains our proposed heuristic method. An explanation of the mechanism is provided here regarding the proposed method and its algorithm. Section 4 shows the experimental evaluations by applying our proposed method to five actual CNN models. Lastly, we provide a conclusion of the paper.

## 2. Background and Definitions

### 2.1. Object Detection Methods by CNNs

Object detection has been a long-standing and fundamental research theme in the computer vision field that detects object instances of a certain class such as a person, animal or vehicle in digital images. The object detection technique has evolved over the past 50 years. Since Krizhevsky [8] won the ImageNet Large Scale Visual Recognition Challenge (ILSVRC) competition in 2012 with a CNN model called AlexNet, CNN-based methods have gradually become popular in object detection applications. In the past few decades, the CNN-based method has been investigating as the mainstream approach in the field of object detection. In the last decade, many methods and algorithms have been aggressively proposed by researchers. For example, we can find the famous R-CNN series [4] and YOLO ones [6] that show excellent performance in their object detection ability. In addition, VGGNET [23], ResNet [24] and DenseNet [25] also demonstrated remarkable performance with deeper architectures.

**Figure 1:**
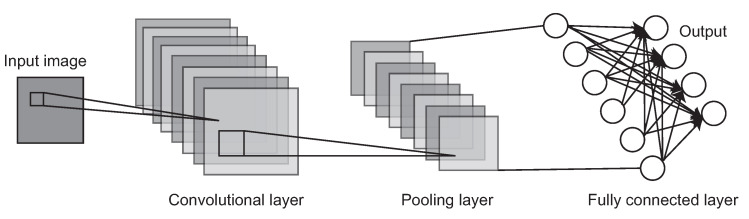
A basic architecture of the Convolutional Neural Network (CNN). A typical CNN is mainly composed of a convolutional layer, a pooling layer and a fully connected layer.

Figure 1 shows the basic architecture of a convolutional neural network. Typically, there are two main parts in the CNN architecture: the convolutional layer and the fully connected layer. The convolutional layer is the key component of the CNN architecture, consisting of a group of filters. By performing massive operation of the convolution, these filters can extract features from the input image. By applying multiple convolutional layers, the CNN can detect various objects such as persons, animals, or vehicles. The fully connected layer is the most elementary component of a CNN architecture, which consists of a string of neurons. By using the output features from the convolutional layers, the CNN can classify the objects in the input image based on the datasets, and finally output the results as numerical likelihood of images.

However, along with their remarkable improvement on performance, the state-of-the-art CNNs always have more complicated architectures. In other words, there are multiple convolutional layers in such CNNs. These cause a huge increase in the convolution operations and the number of parameters [26]. Since the greatest computational overhead of CNN is related to the convolutional layers, it is necessary to accelerate the computation using a hardware accelerator. To overcome the overhead issue, there are three options of the accelerators for the implementation of the convolution process in CNN: CPUs (central processing units) that include multiply-and-add instructions [27,28,29], GPUs (graphics processing units) that execute massively parallel operations [30,31], and FPGAs (field programmable gate arrays) that implement multiple operators in hardware [32,33]. Furthermore, with the increasing number of parameters in the fully connected layer of CNNs, the model size is growing significantly. This will lead to an exponential increase in computational cost and required memory size. In the CPU-based implementations, it is difficult to manage the necessary computations on the resources. On the other hand, in the GPU-based implementations, the increasing number of PEs (processing elements) also leads to a great requirement for memory size. Additionally, the current industry-favored GPUs are extremely depriving the computational power for inference by CNN [34]. This means that the platform needs to integrate more PEs and increase the clock frequency. This will bring higher power consumption. Therefore, we need some technique to provide greater energy-efficiency for the platform. In comparison to GPUs, FPGAs are considered an energy-efficient platform for the CNN. However, FPGAs can have relatively limited amount of memory and hardware resources for the computation. Thus, it is a critical problem to minimize the CNN model before implementation while maintaining high accuracy.

### 2.2. Object Detection Methods by CNNs

As one of the typical model compression methods, network pruning has been widely studied owing to its remarkable performance improvements for the inference process and high compatibility with the original network. Network pruning reduces the size of CNN models by removing unnecessary components from the CNN models while maintaining accuracy for the inference.

The major branch of the network pruning method is called unstructured pruning, which is also known as weight pruning since its main focus of the technique is to remove the redundant weights. It has been studied since its appearance in the 1990s [21]. Recently, Han et al. [35] proposed a method based on the magnitude of the weight. It achieves a high compression ratio by removing the weights beneath a certain threshold of the pruning ratio. The method is further incorporated into another technique known as deep compression, reported in 2016 [22], that achieves an efficient implementation.

Structured pruning is another branch of the network pruning method. It compresses the models through removing redundant structures such as channels, filters or layers. Among existing structured pruning methods, channel pruning is considered as the most effective for compressing the model while maintaining the accuracy for inference. Since the channel is the most fine-grained structured level, it can achieve a higher compression ratio compared to the other types of structured pruning methods. Many advanced research works focused on the channel pruning approach have been presented. Liu et al. proposed network slimming [19], which imposes sparsity regularization on the scaling factors associated with each channel in convolutional layers. Then, the method removed these channels with small scaling factor values. In the same year, Luo et al. [20] introduced a method through a minimization of the feature reconstruction error in the subsequent layer to decide which channels are to be kept. Recently, with the great development of the automated machine learning called AutoML technique, many recent network pruning methods have applied the AutoML to hyperparameter determination. The hyperparameters are used for determining which network structures are to be removed. The latest research proposed by He et al. [36] in 2018 introduced a method that selects unimportant channels to be removed.

In conclusion, we summarize the discussion above into two directions for the compression techniques of the CNN models. One is to reduce the amount of computations by reducing the number of convolutional layers. Then, we can compress the network structure further by using the structured pruning approach. The other direction is to reduce the network size while maintaining accuracy by reducing the number of fan-ins to each node in the middle layers of the fully connected layers. This can be addressed via the unstructured pruning methods.

In this paper, for the structure pruning, we focus on network slimming. For the unstructured pruning, we focus on the weight pruning method utilized in the deep compression.

**Figure 2:**
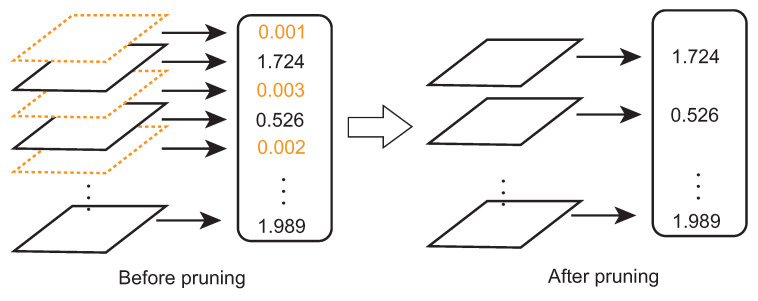
The network slimming process. The channels (left side in orange color) with small scaling factor values (the numbers in orange color) will be eliminated.

**Network Slimming.** Figure 2 illustrates the steps of the network slimming. A channel-associated scaling factor is introduced in this method. The scaling factor is originally defined in [37] as the scale parameters for each batch normalization (BN) layer. As the modern CNN models always adopt a BN layer right after a convolutional layer, the scaling factors in BN layers can be directly leveraged for identifying the unimportant channels. While L1 regularization on scaling factors in the convolutional layers is imposed, the values of scaling factors corresponding to the unnecessary convolutional channels are pushed towards zero. The channels with small values (below a pre-defined threshold) of the scaling factors will be removed as shown in the figure on the left side in orange color. Through the above process, the CNN models can be efficiently compressed especially regarding the convolutional layers.

**Figure 3:**
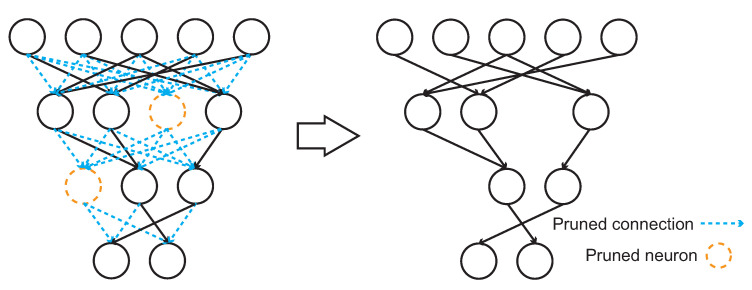
The weight pruning process. The connections (left side in blue color) among neurons with small weight values will be eliminated. The neurons (left side in orange color) without any input or output connection will also be eliminated.

**Deep Compression.** Figure 3 shows a process of the weight pruning method used in the deep compression. It removes the connections shown in the left-side figure in blue color between neurons with weight values beneath a given threshold from the network structure. Moreover, during the reduction of connections, it also removes the neurons in orange color of the left-side figure without input or output connections. Due to the reduction of parameters (i.e., weights) especially for the dense parameters in the fully connected layers, the method finally achieves the compressed model for a CNN.

Therefore, by combining the structured pruning method related to the convolution layers and the unstructured one for the fully connected layer, it is possible to achieve a higher compression ratio than the case employing individual use of these methods, respectively. Additionally, due to the use of these two approaches during a pruning process, we can gain merits from both the structured and the unstructured techniques. This approach can finally prune the entire structure of a CNN model focusing on both the convolutional and fully connected layers. However, because these two methods can influence each other between the convolutional and fully connected layers, it is not clear on how to decide the order of the two methods to be applied to a CNN model and/or the pruning ratios configured to each method in combining the two methods. Thus, in this paper, we will propose a new method that implements an appropriate combination of these two network pruning methods to find the minimized model with a given accuracy for the inference.

## 3. Heuristic Method for Minimizing Model Size of CNN

### 3.1. Strategy for Minimizing the Model Size

Let us present our proposed method in detail including the pruning scheme and the approach for the compression. We setup the approach to achieve the best compression ratio with the structured and unstructured pruning methods. We also introduce the parameters in our proposed method to be defined by application.

**Pruning scheme.** We first apply the structured pruning method to a CNN model to compress the convolutional layers. The reduction ratio is gradually increased, and each ratio is applied to the original network until the model accuracy reaches beneath a given target accuracy. Here, the reduction ratio is defined as the percentage for compression given to the pruning process. Then, the pruning method switches to the unstructured pruning method for compressing the fully connected layers while also increasing the reduction ratio until the accuracy reaches beneath the target accuracy again. Through these two pruning methods, we can finally identify the model that is minimized in size. As we express mathematically above, assume SP(m,p) is a compression of the structured pruning for a model *m* in a percentage of the reduction ratio *p*. It returns a pair of the compressed model $m^{\prime }$ and the accuracy Acc. By increasing *p* and comparing Acc with the target accuracy, $m^{\prime }$ is passed to the unstructured pruning $USP(m^{\prime },p^{\prime })$ when Acc is less than the target accuracy. The unstructured pruning also returns the compressed model $m^{\prime \prime }$ and the accuracy $Acc^{\prime }$. Finally, by increasing the reduction ratio $p^{\prime }$ and comparing $Acc^{\prime }$ with the target accuracy, it finishes the compression at the reduction ratio right before $p^{\prime }$ results in worse accuracy than the target one.

**Initial Margin.** As illustrated above, the structured pruning method compresses a CNN model with every reduction ratio gradually increased until the accuracy does not meet the target one. The compressed model gained after the pruning process is minimized by this structured pruning. Here, if we directly pass the compressed model to the unstructured pruning method, it might not leave enough room for further compression by the unstructured pruning method. Therefore, our method introduces a margin for the reduction ratio after the former pruning method when $m^{\prime }$ is passed to the unstructured pruning. The reduction ratio *p* when $m^{\prime }$ is derived is drawn back for an amount of the margin value before the pruning method is switched to unstructured pruning.

**Parameters to be configured.** There are three parameters defined for our proposed method. The parameters are defined by application side. The first one is the target accuracy for the final compressed model. It affects the size of the final compressed model. The second one is the step_width to be used for increasing the reduction ratios *p* and $p^{\prime }$ for the structured and the unstructured pruning methods. A bigger step_width will help us accelerate the compression. However, it might miss the reduction ratio that achieves the minimum size of the compressed model because it passes over the reduction ratio by the large step_width used for incrementing the ratio. On the other hand, when the step_width is too small, we inevitably need to invoke a large number of iterations to find the minimized compressed model. This will result in a waste of calculation resources and execution time. The final parameter is the margin. As we mentioned above, the margin is used for finding a suitable reduction ratio for the structured pruning method. Hence, if the margin is configured to a large value, our method switches to invoke the unstructured pruning method to reduce the model size that is not compressed adequately by the structured pruning phase. In addition, when the margin is small, the model would be over-compressed by the structured pruning as it is difficult for further compression through the unstructured pruning.

**Figure 4:**
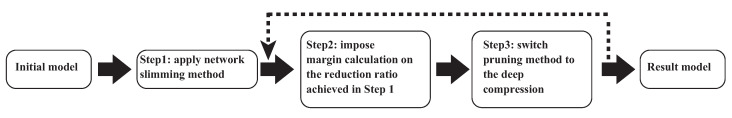
A flow chart of the three-step procedure of our proposed method.

**Figure 5:**
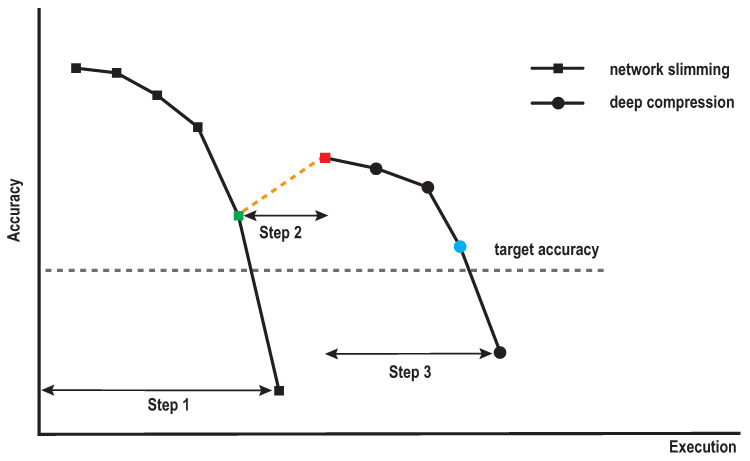
Compression iterations of our proposed method during the search process for the minimal accuracy. Step 1 is the iteration to find the minimal model by structured pruning. Step 2 is the iteration defined by the margin. Step 3 is the iteration to find the minimal model by the unstructured pruning.

Our proposed method employs the three-step procedure as shown in Figure 4. Figure 5 illustrates the accuracy changes in every execution of two pruning methods during the procedure. **Step 1** applies network slimming to the initial model until the model’s accuracy becomes beneath the target accuracy (dotted line in gray color). **Step 2** draws back (dotted line in yellow color) the reduction ratio during Step 1 according to the margin value (the square in red color) for obtaining the model that can be further pruned (the square in green color). **Step 3** switches to the deep compression until the accuracy becomes beneath the target accuracy again. Here, we achieve the reduction ratio derived in the previous accuracy (the circle in blue color) for the deep compression. At the end, our method returns the minimum model derived from the Step 3. In addition, we will repeat Step 2 and Step 3 with different margin values for minimizing the model size. Through the above process, the convolutional layers and the fully connected layers will be effectively compressed. Furthermore, thus, we obtain a model that is minimized in size.

### 3.2. Algorithms for Minimizing the Model Size

To summarize the overall mechanism, we show the computation flow of the proposed method in Algorithm 1. The algorithm first loads the pre-trained model init_model as the input for the algorithm. It initializes the reduction ratios of two pruning methods of the network slimming NS_ratio and the deep compression DC_ratio to the corresponding step_width values (lines 1–2). The lines 3–4 describe the procedures of Step 1 in Figure 5 while iterating the network slimming until the model’s accuracy Acc becomes lower than the target accuracy Acc_th. The algorithm derives the reduction ratio for the network slimming method in NS_ratio. Here, the model NS_model derived from the iterations above is the minimal compressed model by the network slimming. The lines 8–9 correspond to Step 2 in Figure 5 where the procedure relates to the margin. Through the margin calculation on this reduction ratio, we obtain a compressed model drawn back due to the margin value and the algorithm updates NS_model to the model. The lines 10–12 correspond to Step 3 in Figure 5 iterating the deep compression until the model accuracy Acc becomes lower than the target accuracy acc_th again. Thus, the minimal model is derived. Finally, the algorithm returns the final_model as the output of the compression process (line 15). Moreover, we also assign multiple values to the margin by gradually decreasing the initial_margin. Furthermore, through repeating the procedures of lines 8–13, we will achieve the model final_model as the minimized model regarding the size.

Here, let us consider the elapsed time of our proposed method. We define the valuables related to the execution time of the algorithm as follows. *N* is the number of iterations in lines 8–13 of the algorithm. t1 is the average time for the network slimming. τ1 is the number of iterations in lines 3–4. t2 is the average time of the deep compression. τ2 is the number of iterations of the deep compression in lines 10–11. The elapsed time of our algorithm is calculated as follows:

Elapsed time=t1×τ1+N×(t1+t2×τ2)

For investigating the efficiency of our algorithm, let us compare it with the brute-force search method to find the minimized model. The elapsed time is defined as follows:

$$Elapsed\;time=\frac{100}{step\_width}\times t1\times \frac{100}{step\_width}\times t2$$

**Algorithm 1** Pseudocode of algorithm for Minimizing Model Size

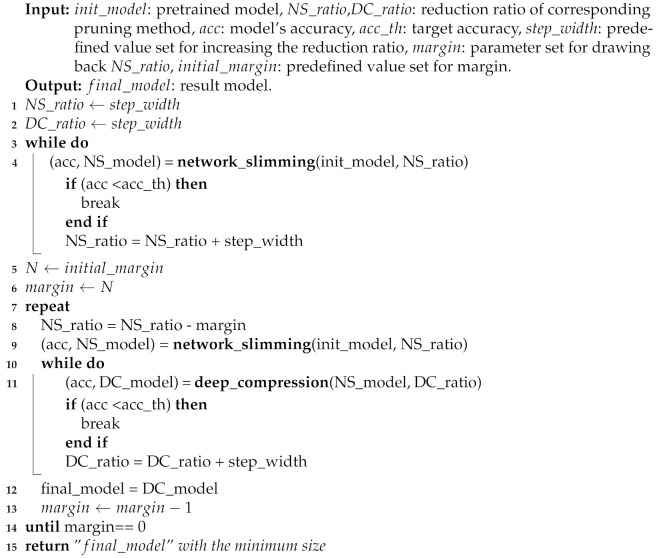



Here, the step_width is the value to define the incremental step_width for the reduction ratio during the structured and the unstructured pruning methods. If we increase the reduction ratio at every percentage as an integer value, the step_width becomes 1. Therefore, the elapsed time becomes 10,000×t1×t2. In our method, we should configure τ1<100, τ2<100 and N<100. Thus, it is clear that our proposed method will achieve less execution time than the brute-force search approach.

Through the procedures above, our proposed method will utilize the structured pruning method for focusing on compressing the convolutional layers, and then, the unstructured one for reducing the fully connected layers. In comparison with the other existing approaches, our method not only removes the unnecessary network structures but also directly acts on the reduction of unimportant connections and neurons by utilizing unstructured pruning.

In summary, network slimming is used as the structured pruning in our work, which compresses the convolutional layers by removing the unimportant channels. For the unstructured pruning, we take advantage of the deep compression. It works especially well on the fully connected layers where the greater part of the parameters of the entire network is concentrated.

## 4. Experimental Evaluations

### 4.1. Experimental Setup

Now, we discuss the performance of our proposed method. We evaluate the performance of our proposed method with several state-of-the-art CNN models, including VGGNet, ResNet and DenseNet, on the CIFAR-10 dataset. The results of the evaluations will be discussed from three aspects: model size, calculation overhead and execution time.

For our experiment, we use a variation of the VGG-19 with 16 convolutional layers and 1 fully connected layer. For Resnet, a 110-layer pre-activation Resnet with bottleneck structure (denoted as ResNet-110) is used. For DenseNet, we experiment with a 40-layer DenseNet (denoted as DenseNet-40), a 121-layer DenseNet (denoted as DenseNet-121) and a 201-layer DenseNet (DenseNet-201), respectively. Furthermore, for all models, we adopt the network model with batch normalization layer from [37]. The model size and accuracy of these CNN models are illustrated in Table 1. The dataset we select is the CIFAR-10 [38] dataset, which consists of a training set of 50,000 32×32 color images and a testing set of 10,000 32×32 color images. We use it for training the original CNN models and testing the result models as well. For the comparison of the results, we show the compression performances when we apply network slimming and deep compression individually. The comparing results are derived by these individual methods to achieve the same target accuracies used for evaluating our method. In the following evaluations, we perform experiments that analyze the effects of our method in the compressed model size and the processing time to reach the minimum size, respectively. Then, finally, we discuss the validity of our method focusing on the heuristic approach.

During the performance evaluations below, we implemented the compression system of Algorithm 1 on a GPU-based environment by using the state-of-the-art network models with the CIFAR-10 dataset. Our proposed method is implemented with PyTorch framework. The experiments are performed on a computer using Linux Ubuntu 18.04 operating system with Intel Xeon E5-2698 V4 CPU, 512GB RAM, and one NVIDIA Tesla V100 with 32 GB VRAM.

### 4.2. Evaluation on Minimizing Performance for Model Size

We first evaluate the minimizing performance for model size. Besides performing our method on each CNN model, we also individually implement network slimming and deep compression on these models. According to the original accuracy of the five CNN models, we set three target accuracies for each model. The detailed results of each CNN model are presented in Table 2, Table 3, Table 4, Table 5 and Table 6, respectively. We also show the margin at which the minimum size has been obtained in the tables (the number in the brackets in the column ‘Ours’) under each accuracy condition for the five CNN models.

The result for VGG-19 is summarized in Table 2. When the target accuracy varies among 85%, 90% and 95%, the result model size of our proposed method becomes 5.91 MB, 6.11 MB and 6.23 MB, respectively. The pruned ratio (the ratio reduced from the original size) also becomes 92.6%, 92.4% and 92.2%, respectively. Although the pruned ratio decreased against the increase of the target accuracy, our method remains the best pruned ratio compared to the individual pruning of the deep compression and network slimming. In addition, we can see that the deep compression method performs better than the network slimming method, with the result model size of 6.42 MB (target accuracy: 85%), 7.23 MB (target accuracy: 90%) and 7.23 MB (target accuracy: 95%), while the network slimming method was able to reduce until 10.2 MB. This is due to the fact that the deep compression method can remove more parameters from the parameter-intensive fully connected layers than the network slimming.

The experimental results for the ResNet-110 and DenseNet-40 models are presented in Table 3 and Table 4. Although the baseline model sizes of the network slimming and deep compression were only 4.61 MB (ResNet-110) and 4.26 MB (DenseNet-40), our proposed method can still be well applied. Corresponding to the given target accuracy, for ResNet-110, we achieved the pruned ratios of 75.5%, 68.8% and 53.4% regarding each target accuracy. For DenseNet-40, the pruned ratios have become 84.0%, 78.9%, 73.7% for each target accuracy, respectively. Compared to the case where the individual pruning method of network slimming and deep compression is applied, respectively, our proposed method still maintains the best pruned ratio.

Furthermore, in the case of DenseNet-121 and DenseNet-201 applied to the CNN models that have complex and deeper network structures, our method also achieves the best compression ratio as listed in Table 5 and Table 6. Here, DenseNet-201 is the largest original model size among the five experiment models. When the target accuracy was varied among 85%, 90% and 95%, our proposed method obtained the result model sizes with 3.52 MB, 4.32 MB and 6.87 MB, respectively. The pruned ratio even reached 97.0% with the given target accuracy of 85%. Furthermore, for DenseNet-121, the pruned ratio also achieved more than 90%.

On the other hand, we notice that both our method and the deep compression perform much better than the network slimming method as shown in Table 3, Table 4, Table 5 and Table 6. Especially for the ResNet-100 model, our method and the deep compression achieved the pruned ratio above 50% while the pruned ratio of the network slimming achieved below 20%. This demonstrates that after the network slimming finishes removing the unimportant channels beneath the given threshold of the accuracy, the deep compression can still compress the network focusing on the level of weights.

It has been confirmed that our method can minimize the model size compared with cases where network slimming and deep compression are applied individually.

### 4.3. Evaluation for Calculation Overhead and Execution Time

We further evaluate the calculation overhead and the execution time. The execution times of each CNN model with pre-defined target accuracies are shown in Table 7, Table 8, Table 9, Table 10 and Table 11. Since the execution time is mainly consumed during the accuracy check by comparing the pruned model’s accuracy with the target accuracy, we also show the number of checks in the tables (the number in the bracket of the column ‘Ours’). The execution time for a brute-force search method is also shown. In our experiment, the brute-force search method tries to find the minimum model size through every compression trial while varying the reduction ratios of network slimming and deep compression incrementally from 1 to 100, respectively. This means, in the brute-force search method, pruning needs to be executed 10,000 times to find the minimum model. Therefore, the execution time required has become very large as shown in Table 7, Table 8, Table 9, Table 10 and Table 11.

As we can see in lines 7–15 shown in Algorithm 1, the number of iterations in lines 8–13 that occupy the largest part of the execution time is affected by the value of the initial margin. Here, we assigned the initial margin to 50. The execution time of our proposed method for each model is presented in Table 7, Table 8, Table 9, Table 10 and Table 11.

Comparing the results of our method with the brute-force search method, focusing on VGG-19 as shown in Table 7, our method reduces the number of accuracy checks 15-fold and is 15 times faster regarding all target accuracy conditions. For ResNet-110, in the case where the target accuracy is 94%, the execution time achieves a 28-fold increase in speed and a 26-fold reduction in the number of accuracy checks as illustrated in Table 8. Table 9 shows the result for DenseNet-40. It achieves a 15-fold increase in speed. The results for DenseNet-121 and DenseNet-202 are demonstrated in Table 10 and Table 11. The results of the network slimming and deep compression maintained higher accuracy through the deeper network structures. In other words, more computation-intensive convolutional layers are utilized in these models. Therefore, the execution time was reduced drastically. As shown in the tables, regarding the brute-force search method, the execution time even reached 28 h and 92.5 h. Our method still achieves 14- and 15-fold increases in speed. Moreover, the number of accuracy checks for these two models is also reduced 14-fold compared to the brute-force search method.

Overall, through the results shown in Table 7, Table 8, Table 9, Table 10 and Table 11, we can conclude that our proposed method derives the minimized model with less computational cost. Thus, our method will find the minimum model in the shortest execution time.

## 5. Discussion

In this section, we discuss the order of the pruning methods used in our proposed method. Our proposed approach tries to minimize the model size by combining two kinds of pruning methods. In order to maximize the benefits of the two pruning methods, we conduct a further experiment to concretely establish the effective execution order of two pruning methods. We try to compress the five models in order from deep compression to network slimming (abbreviated as DC→NS). Then, we compare the compression ratios with the ones achieved by the order (abbreviated as NS→DC) used in our method. Table 12, Table 13, Table 14, Table 15 and Table 16 show the compression ratios of NS only, DC only, DC→NS and NS→DC for the five models under given target accuracies.

Table 12 shows the results for VGG-19 when the sequence is DC→NS. We achieved the minimized model that was over 20% better than the case of the individual execution of the network slimming. However, the result shows about 10% worse model size than the deep compression. The order NS→DC performs the best compression ratio compared with those of DC→NS, deep compression and network slimming. According to Table 13 and Table 14, for ResNet-110 and DenseNet-40, all cases except the one of NS only obtained similar compressed model sizes. However, under all conditions of the target accuracies, the cases of NS→DC remain the highest pruned ratio even though they are only a few percentage points higher than the other cases. Based on the results of DenseNet-121 and DenseNet-202 presented in Table 15 and Table 16, it has been confirmed that the NS→DC also achieved the minimum model size. When the target accuracy is 90%, both DenseNet-121 and DenseNet-202, in the case of NS→DC performs 16% better than the one of the DC→NS in the minimized model size. Therefore, we heuristically choose NS→DC as the execution order for two pruning methods in our proposed approach.

**Figure 6:**
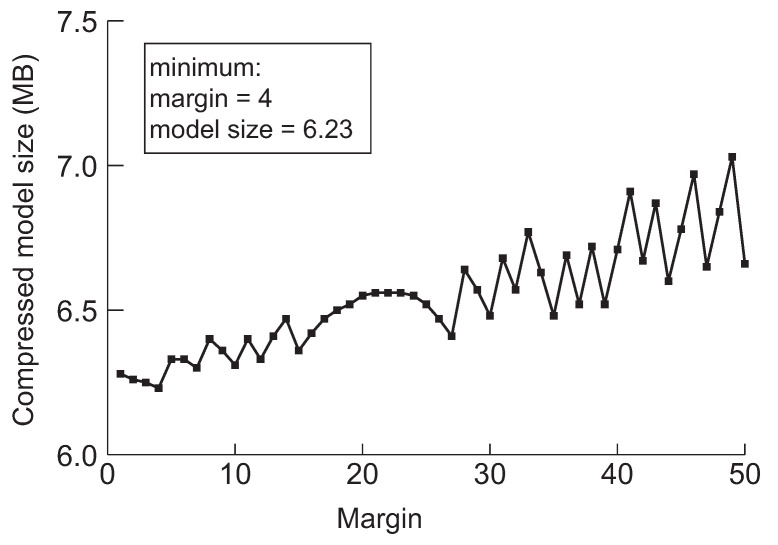
The minimum model sizes derived by the proposed method when the initial margin is varied from 1 to 50 for VGG-19 when the target accuracy is 92%.

**Figure 7:**
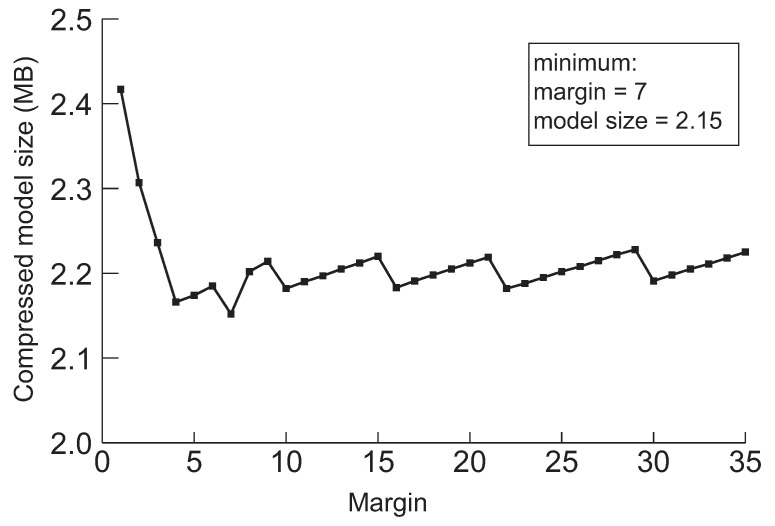
The minimum model sizes derived by the proposed method when the initial margin is varied from 1 to 50 for ResNet-110 when the target accuracy is 94%.

**Figure 8:**
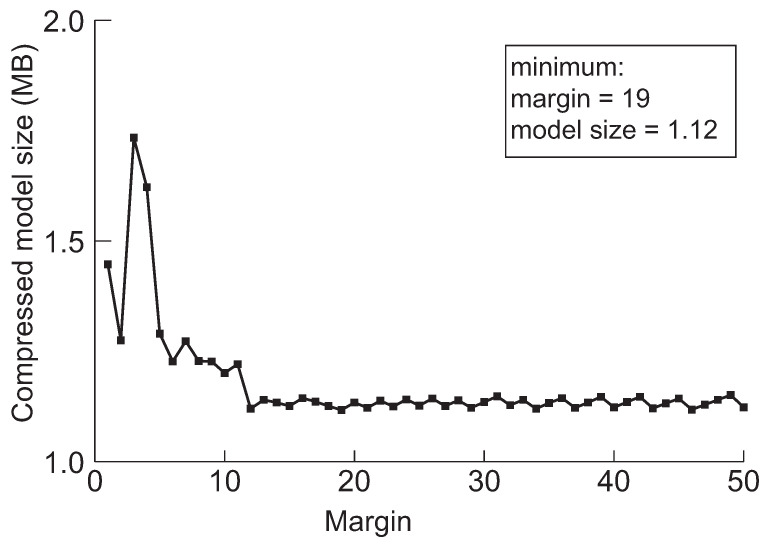
The minimum model sizes derived by the proposed method when the initial margin is varied from 1 to 50 for DenseNet-40 when the target accuracy is 92%.

**Figure 9:**
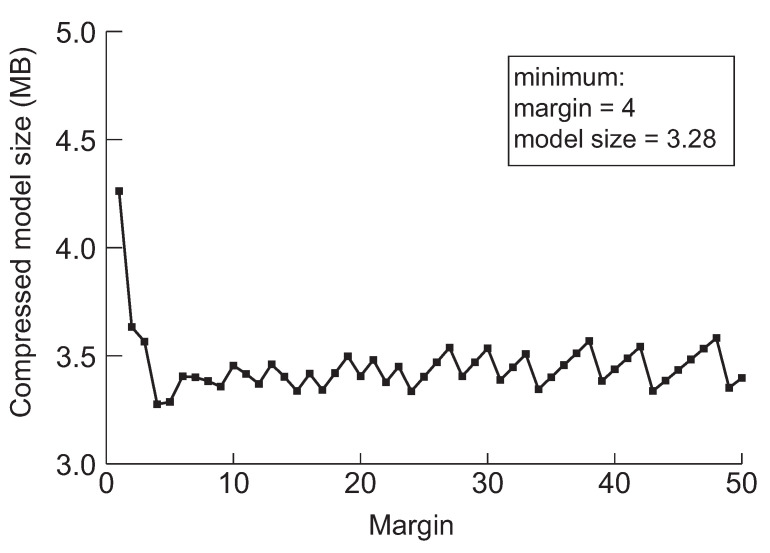
The minimum model sizes derived by the proposed method when the initial margin is varied from 1 to 50 for DenseNet-121 when the target accuracy is 94%.

**Figure 10:**
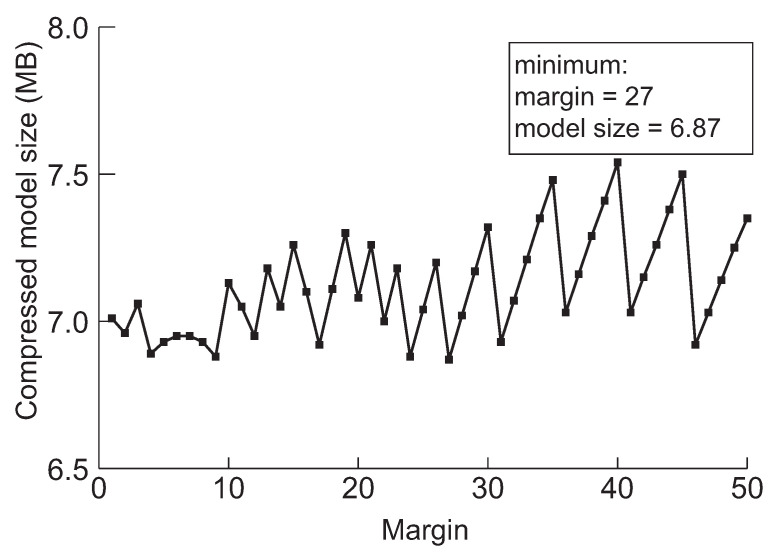
The minimum model sizes derived by the proposed method when the initial margin is varied from 1 to 50 for DenseNet-202 when the target accuracy is 95%.

Next, we discuss a crucial parameter in our approach, the margin. The margin is used for drawing back the model before the pruning method switches from network slimming to deep compression. Furthermore, the purpose of the initial margin is to control the number of iterations for finding the minimum model by performing further pruning. The model will be over-compressed by network slimming if we draw back too little, and then, it will be difficult to perform further compression through the deep compression. We experimented to minimize the five models applying the margin value while varying from 1 to 50 to evaluate the effect of drawing back during searching the minimum model size. The results are summarized in Figure 6, Figure 7, Figure 8, Figure 9 and Figure 10, respectively.

According to the experimental results in the cases of VGG-19, ResNet-110, DenseNet-40, DenseNet-121 and DenseNet-202 shown in Figure 6, Figure 7, Figure 8, Figure 9 and Figure 10, respectively, it has been confirmed that our method can finally derive the minimized model with the margin calculation by repeating deep compression and checking the model size. The result of VGG-19 in Figure 6 shows that the minimum model was found when the value of margin was 4. The minimum size of the model was 6.23 MB. Additionally, in the case of ResNet-110, the value for the margin was 7 when the model size was minimized as shown in Figure 8. Our method found the minimum model size for DenseNet-40, DenseNet-121 and DenseNet-202 while the margin was configured to 19, 4 and 27, respectively. Based on the results shown in Figure 6, Figure 7, Figure 8, Figure 9 and Figure 10, we thus conclude that each of the five models achieved the minimum model size while the margin maintained a value below 50. Therefore, we heuristically determined the initial margin to be 50 in this paper.

## 6. Conclusions

This paper describes a heuristic method for minimizing the model size of CNN by combining the structured pruning and unstructured pruning methods. Compared with the performances in which the pruning methods are applied individually, our method takes advantage of the structured pruning method for compressing the convolutional layers and also removes the dense parameters in the fully connected layers through the unstructured pruning method. The results of evaluations using five CNN models showed that our method could find the minimized model while satisfying the given target accuracy. Furthermore, our method drastically reduces the runtime and computing operations compared with the brute-force search method.

For future work, we will apply our proposed method to various CNN models to find the minimum model sizes under given target accuracies. Additionally, we plan to extend our method by combining other compression techniques such as quantization, low rank, or distillation and identify the most effective combination for minimizing the model size.

## Figures and Tables

**Table 1:** The original model size and accuracies of CNN models used in experiments.

Model	Accuracy	Model Size
VGG-19	93.99%	80.34 MB
ResNet-110	94.59%	4.61 MB
DenseNet-40	94.16%	4.26 MB
DenseNet-121	95.51%	42.15 MB
DenseNet-202	95.99%	117.24 MB

**Table 2:** The sizes of the minimized models in the corresponding accuracies derived from the proposed method and the individual cases of network slimming and deep compression for VGG-19.

Target Accuracy	Deep Compression	Network Slimming	Ours (Margin)
85%	6.42 MB	10.2 MB	5.91 MB (4)
90%	7.23 MB	10.2 MB	6.11 MB (5)
92%	7.23 MB	10.2 MB	6.23 MB (4)

**Table 3:** The sizes of the minimized models in the corresponding accuracies derived from the proposed method and the individual cases of network slimming and deep compression for ResNet-110.

Target Accuracy	Deep Compression	Network Slimming	Ours (Margin)
85%	1.14 MB	3.77 MB	1.13 MB (20)
90%	1.46 MB	3.87 MB	1.44 MB (40)
94%	2.19 MB	4.06 MB	2.15 MB (7)

**Table 4:** The sizes of the minimized models in the corresponding accuracies derived from the proposed method and the individual cases of network slimming and deep compression for DenseNet-40.

Target Accuracy	Deep Compression	Network Slimming	Ours (Margin)
85%	0.71 MB	1.81 MB	0.68 MB (21)
90%	0.92 MB	1.97 MB	0.90 MB (16)
92%	1.13 MB	2.02 MB	1.12 MB (19)

**Table 5:** The sizes of the minimized models in the corresponding accuracies derived from the proposed method and the individual cases of network slimming and deep compression for DenseNet-121.

Target Accuracy	Deep Compression	Network Slimming	Ours (Margin)
85%	2.49 MB	8.30 MB	2.28 MB (22)
90%	2.90 MB	8.72 MB	2.49 MB (25)
94%	3.73 MB	9.96 MB	3.73 MB (4)

**Table 6:** The sizes of the minimized models in the corresponding accuracies derived from the proposed method and the individual cases of network slimming and deep compression for DenseNet-202.

Target Accuracy	Deep Compression	Network Slimming	Ours (Margin)
85%	4.69 MB	15.10 MB	3.52 MB (50)
90%	4.69 MB	15.10 MB	4.32 MB (29)
95%	7.03 MB	19.07 MB	6.87 MB (27)

**Table 7:** The execution times in the corresponding accuracies derived from the proposed method and the individual cases of network slimming and deep compression for VGG-19.

Target Accuracy	Brute-Force Search Method	Ours (Number of Accuracy Check)
85%	7.5 h	0.5 h (656)
90%	7.5 h	0.5 h (659)
92%	7.5 h	0.5 h (654)

**Table 8:** The execution times in the corresponding accuracies derived from the proposed method and the individual cases of network slimming and deep compression for ResNet-110.

Target Accuracy	Brute-Force Search Method	Ours (Number of Accuracy Check)
85%	8.3 h	0.45 h (597)
90%	8.3 h	0.35 h (470)
94%	8.3 h	0.30 h (388)

**Table 9:** The execution times in the corresponding accuracies derived from the proposed method and the individual cases of network slimming and deep compression for DenseNet-40.

Target Accuracy	Brute-Force Search Method	Ours (Number of Accuracy Check)
85%	7.5 h	0.6 h (663)
90%	7.5 h	0.6 h (678)
92%	7.5 h	0.5 h (579)

**Table 10:** The execution times in the corresponding accuracies derived from the proposed method and the individual cases of network slimming and deep compression for DenseNet-121.

Target Accuracy	Brute-Force Search Method	Ours (Number of Accuracy Check)
85%	28 h	2.1 h (737)
90%	28 h	1.9 h (690)
94%	28 h	2.0 h (705)

**Table 11:** The execution times in the corresponding accuracies derived from the proposed method and the individual cases of network slimming and deep compression for DenseNet 202.

Target Accuracy	Brute-Force Search Method	Ours (Number of Accuracy Check)
85%	92.5 h	6.3 h (689)
90%	92.5 h	6.35 h (719)
95%	92.5 h	6.5 h (739)

**Table 12:** The sizes of the minimized models in the corresponding accuracies derived from the individual cases of network slimming and deep compression, NS→DC and DC→NS for VGG-19.

Target Accuracy	Deep Compression	Network Slimming	NS→DC	DC→NS
85%	6.42 MB	10.2 MB	5.91 MB	7.23 MB
90%	7.23 MB	10.2 MB	6.11 MB	8.03 MB
92%	7.23 MB	10.2 MB	6.23 MB	8.03 MB

**Table 13:** The sizes of the minimized models in the corresponding accuracies derived from the individual cases of network slimming and deep compression, NS→DC and DC→NS for ResNet-110.

Target Accuracy	Deep Compression	Network Slimming	NS→DC	DC→NS
85%	1.14 MB	3.77 MB	1.13 MB	1.17 MB
90%	1.46 MB	3.87 MB	1.44 MB	1.47 MB
94%	2.19 MB	4.06 MB	2.15 MB	2.19 MB

**Table 14:** The sizes of the minimized models in the corresponding accuracies derived from the individual cases of network slimming and deep compression, NS→DC and DC→NS for DenseNet-40.

Target Accuracy	Deep Compression	Network Slimming	NS→DC	DC→NS
85%	0.71 MB	1.81 MB	0.68 MB	0.73 MB
90%	0.92 MB	1.97 MB	0.90 MB	0.94 MB
92%	1.13 MB	2.02 MB	1.12 MB	1.14 MB

**Table 15:** The sizes of the minimized models in the corresponding accuracies derived from the individual cases of network slimming and deep compression, NS→DC and DC→NS for DenseNet-121.

Target Accuracy	Deep Compression	Network Slimming	NS→DC	DC→NS
85%	2.49 MB	8.30 MB	2.28 MB	2.55 MB
90%	2.90 MB	8.72 MB	2.49 MB	2.96 MB
94%	3.73 MB	9.96 MB	3.28 MB	3.89 MB

**Table 16:** The sizes of the minimized models in the corresponding accuracies derived from the individual cases of network slimming and deep compression, NS→DC and DC→NS for DenseNet-202.

Target Accuracy	Deep Compression	Network Slimming	NS→DC	DC→NS
85%	4.69 MB	15.09 MB	3.52 MB	4.84 MB
90%	4.69 MB	15.09 MB	4.32 MB	5.15 MB
95%	7.03 MB	19.07 MB	6.87 MB	8.21 MB

## Data Availability

Not applicable.
